# Fabrication of Soft Biodegradable Foam with Improved Shrinkage Resistance and Thermal Stability

**DOI:** 10.3390/ma17153712

**Published:** 2024-07-27

**Authors:** Fangwei Tian, Hanyi Huang, Yaozong Li, Wentao Zhai

**Affiliations:** School of Materials Science and Engineering, Sun Yat-sen University, Guangzhou 510275, China; tianfw@mail2.sysu.edu.cn (F.T.); huanghanyi@sysunc.com (H.H.); liyz33@mail2.sysu.edu.cn (Y.L.)

**Keywords:** PBAT/PBS, soft foam, shrinkage resistance, thermal stability, physical foaming

## Abstract

The soft PBAT foam shows good flexibility, high elasticity, degradable nature, and it can be used as an environmental-friendly candidate for EVA and PU foams. Unfortunately, there are few reports on the application of PBAT as a soft foam. In this study, PBAT foam was fabricated by a pressure quenching method using CO_2_ as the blowing agent. A significant volume shrinkage of about 81% occurred, where the initial PBAT foam had an extremely high expansion ratio, of about 31 times. A 5–10 wt% PBS with high crystallinity was blended, and N_2_ with low gas solubility and diffusivity was mixed, with the aim of resisting foam shrinkage and preparing PBAT with a high final expansion ratio of 14.7 times. The possible mechanism behind this phenomenon was established, and the increased matrix modulus and decreased pressure difference within and outside the cell structure were the main reasons for the shrinkage resistance. The properties of PBAT and PBAT/PBS foams with a density of 0.1 g/cm^3^ were measured, based on the requirements for shoe applications. The 5–10 wt% PBS loading presented advantages in reducing thermal shrinkage at 75 °C/40 min, without compromising the hardness, elasticity, and the compression set, which ensures that PBAT/PBS foams have good prospects for use as soft foams.

## 1. Introduction

Soft polymeric foams with low density, high resilience, and excellent energy absorption properties find widespread applications in sports footwear [1], automotive [2], sealing, and packaging industries [3,4]. Traditional soft foams, such as ethylene-vinyl acetate copolymer (EVA) and polyurethane (PU) foams are commonly prepared using chemical blowing agents or alkane-based blowing agents [5,6]. However, the foaming process is hazardous and polluting, and the obtained foams exhibit varying degrees of cross-linking, resulting in poor performance, and waste that is difficult to degrade. Recently, the non-cross-linked soft foams such as thermoplastic polyurethane (TPU), thermoplastic polyester elastomer (TPEE), and other physically foamed materials using supercritical fluids as blowing agents have gained popularity [7,8,9,10,11]. These materials possess uniform cell structures, excellent mechanical properties, resilience, environmental benefits, and operational versatility in the foaming process. They are increasingly used as medium and high-end flexible foam alternatives to EVA and other traditional foams [12,13]. Nevertheless, these foam materials are challenging to degrade post-use, posing ongoing environmental pollution risks.

Poly (butylene adipate-co-terephthalate) (PBAT), a biodegradable thermoplastic polyester, is synthesized through the copolymerization of butylene adipate and butylene terephthalate glycols [14]. It demonstrates favorable biodegradability, ductility, and processing properties, thereby exhibiting significant competitive advantages as a substitute for low-density polyethylene (LDPE) in fields such as agriculture, food service, and packaging [15,16,17]. Leveraging these superior attributes, PBAT holds substantial promise for the advancement of soft foams. Researchers have established that PBAT possesses remarkable foamability, successfully fabricating PBAT foams with an expansion ratio ranging from 3 to 20 times and cell sizes between 10 to 100 μm using supercritical carbon dioxide [18]. These foams exhibit commendable flexibility and resilience, positioning them as eco-friendly and sustainable alternatives in the foam industry [19,20]. Regrettably, PBAT has a low melting point (*T*_m_) of 120 °C, low crystallinity, and a low glass transition temperature (*T*_g_). The suboptimal heat resistance and intrinsic shrinkage of PBAT foams under the normal operating temperature pose a significant challenge to the fabrication of high-performance flexible PBAT foams. Meanwhile, the soft matrix makes PBAT foam prone to significant volume shrinkage after foaming, resulting in poor surface quality and difficulties in controlling the dimensional accuracy of the foam [21,22,23]. 

The shrinkage of initial PBAT foams can be reduced through polymer chain modification and chemical structure regulation. Long et al. [24] prepared PBAT foams after the chain branching of PBAT/PLA blends using an epoxy chain extender. They found that the chain-branched PBAT foams could expand up to 22.6 times, and there was no significant foam shrinkage being observed. Unfortunately, the modified PBAT/PLA foams had an increased gel content of about 9.9%, leading to a poor degradation ability [25]. Zhang et al. [26] investigated the influence of chemical structure design on the foaming behavior of PBAT. They found that the butylene terephthalate (BT) content was a critical parameter to dominate the foaming behavior, shrinkage behavior, and degradation characteristics of PBAT. The increment in the BT content from 43% to 61% increased the compression modulus and then the dimensional stability of PBAT foams. At a higher BT content, however, the degradation ability of PBAT was reduced dramatically. 

In addition to the chemical structure design, the polymer blending or compounding are the popular strategies to restrain the foam shrinkage during PBAT foaming. YANG et al. [27] enhanced the shrinkage resistance of PBAT foam by blending with starch. They demonstrated that a 30 wt% starch loading in PBAT could effectively increase structural rigidity and resisted the foam shrinkage of PBAT foam. However, because PBAT and starch were not compatible, the resulting defects reduced the flexibility and spoiled the mechanical properties of PBAT composite foams. HU et al. [28] investigated the effect of PBS blending on the foam expansion and shrinkage of PBAT, and the 20 wt% loading was helpful for the fabrication PBAT foam with zero shrinkage and a high foam expansion ratio of 18.4 times. Unfortunately, a high loading of crystallized PBS reduced the elasticity and flexibility of PBAT foams. Kartik et al. [29] synthesized PBAT nanocomposites by incorporating maleated polypropylene (PP) as a compatibilizer and integrating carbon nanotube (CNT) and organoclay (15A) into the PBAT/PP blend. The incorporation of 3% CNT increased the heat distortion temperature of PBAT composites by 20 °C, indicating improved heat resistance. However, the complex processes, blend compatibility, and degradability may hinder the progress of this research.

Based on the aforementioned studies, the objective of this study is to prepare a lightweight soft PBAT foam with minimal foam shrinkage and increased thermal resistance, as well as to evaluate the possibility of using soft PBAT foam in shoe application. Poly(butylene succinate) (PBS) with 5 and 10 wt% loading was selected to blend with PBAT, since PBS exhibits an excellent degradation ability, high thermal resistance, and a similar chemical structure with that of PBAT. A pressure quenching foaming method was used to foam PBAT and PBAT/PBS blends using CO_2_ and the mixed CO_2_/N_2_ as blowing agents. The influences of N_2_ usage and PBS loading on the foaming behavior and volume shrinkage of PBAT foams were investigated, and a schematic diagram was established to explain the mechanism of foam shrinkage resistance. PBAT and PBAT/PBS blend foams with density of 0.1 g/cm^3^ were fabricated, and the standard properties of soft foam used in shoe application, such as hardness, rebound, compression set, and thermal stability were tested. A comparison of the properties of PBAT foam with those of regular EVA foam and TPU foam was carried out, and the effect of PBS loading on the thermal stability of PBAT foam was discussed. 

## 2. Materials and Methods

### 2.1. Materials

Poly(butylene adipate-co-terephthalate) (PBAT, 2208) raw resin was obtained from Jinghui Zhaolong High-tech Co., Ltd. (Taiyuan, China), with a density of 1.24 g/cm^3^ and a melt mass-flow rate (MFR) and carboxyl content of 4.69 g/10 min and 12.35 Mol/t, respectively. Poly(butylene succinate) (PBS, E810) was provided by Miracll Co., Ltd. (Yantai, China), with a density of 1.25 g/cm^3^ and a melt mass flow rate of 6.54 g/min (190 °C, 2.16 kg). The selected PBAT and PBS have similar melting temperatures of 121 and 114.1 °C, respectively. CO_2_ (purity: 99.9 wt%) and N_2_ (purity: 99.9 wt%) were purchased from Guangzhou Guangqi Gas Co., Ltd., (Guangzhou, China).

### 2.2. Sample Preparation

**Figure 1:**
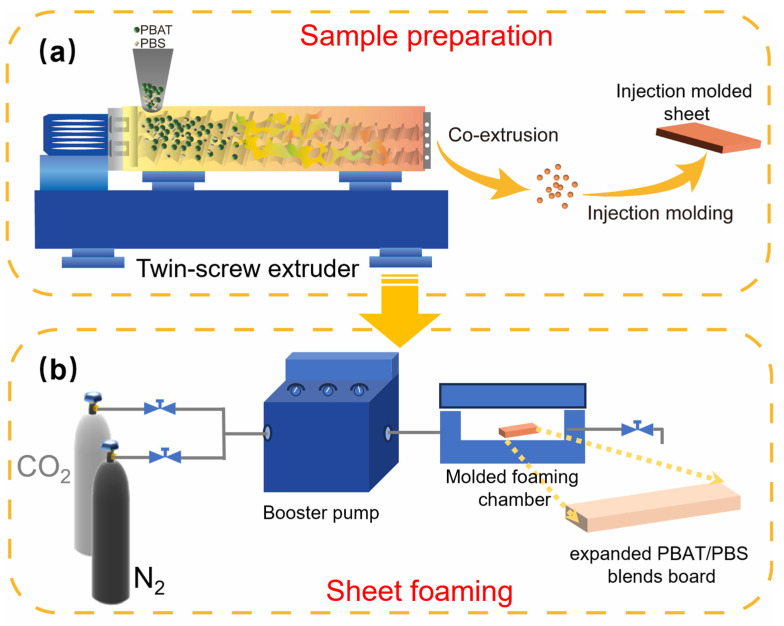
Schematic procedure of PBAT/PBS microcellular foam. (**a**) sheet preparation process; (**b**) sheet foaming process.

Preparation of PBAT/PBS blend composites: PBAT and PBS pellets were pre-dried in a blast oven for 5 h at 80 °C to remove the moisture. Subsequently, the process was carried out according to the process depicted in Figure 1. PBAT/PBS blends were prepared using a twin-screw extruder (Guangzhou POTOP Co., Ltd., Guangzhou, China) at a screw speed of 80 rpm and a temperature of 150 °C. For comparison, pure PBAT was also processed with the same procedure. The contents of PBS were 0 wt%, 5 wt%, and 10 wt%, based on total blends weight. The blends were named PBAT0, PBAT5, and PBAT10, respectively. Injection molded sheets with dimensions of 50 mm × 50 mm × 5 mm were prepared by an injection molding machine (Guangzhou POTOP Co., Ltd., Guangzhou, China) at 12 MPa and 155 °C.

Gas saturation and molded foaming: the PBAT sheets were placed into the sheet foaming chamber with the target temperatures of 102–114 °C. Thereafter, the pressure relief valve was closed and then pressurized with CO_2_ to the target pressure. The valve was then switched to pump in N_2_ until the pressure reached 18 MPa. The samples were treated under this condition for 2 h to ensure equilibrium adsorption of gas. At the end of the saturation, the vessel was released, with a depressurization rate of 10 MPa/s to atmospheric pressure to initiate polymer foaming. During the experiments, the CO_2_ partial pressures were set to 3, 6, 9, and 18 MPa, respectively.

### 2.3. Dispersion Morphology Characterization

The samples were cryogenically fractured after liquid nitrogen immersion for 5 min. After the freeze-cracked surface was sprayed with Au, the PBAT/PBS dispersed phase was observed using a scanning electron microscope (SEM, EM-30, COXEM, Daejeon, Republic of Korea) under the accelerating voltage of 20 kV.

### 2.4. Crystallization Behavior

The crystallization behaviors of PBAT/PBS composites were studied by TA instruments (DSC 250 with TA instruments, New Castle, DE, USA). The samples (3–5 mg) were filled and sealed in an aluminum tray. The whole experiment process was conducted under the protection of a nitrogen atmosphere (flow rate of 20 mL/min). All samples were heated from −50–160 °C at a rate of 10 °C/min (first heating), held isothermal for 3 min, then cooled to −50 °C at a rate of 10 °C/min (first cooling) and finally heated to 160 °C at a rate of 10 °C/min again (second heating). The sample’s degree of crystallinity ($X_{c}$) can be calculated as follows:

(1) $$X_{c}=\frac{∆H_{m}}{∆H_{m0}^{1}\times W_{f}+∆H_{m0}^{2}\times {(1-W}_{f})}\times 100\%$$

where $∆H_{m}$ is the enthalpy of melting, $∆H_{m0}^{1}$ and $∆H_{m0}^{2}$ are the theoretical enthalpy of the 100% crystalline PBAT (114 J/g) an PBS (110.3 J/g) [20,30]. $W_{f}$ is the weight ratio of the PBAT in the PBAT/PBS blends.

### 2.5. Gas Solubility Test

The gas solubility of different gases were tested according to the reported sorption and desorption methods [7,31]. In measurement, the sample was taken out from the chamber after completing a certain saturated temperature and pressure. The weight of the sample over time was measured on a 1 in 100,000 precision balance (AUW120D, Shimadzu, Kyoto, Japan), and the ambient temperature and humidity were 22 °C and 70%, respectively. The duration between the removal of the sample from the container and the measurement of the weight was 10–20 s. The measured data were subsequently fitted to calculate the solubility. It is worth noting that the actual solubility is greater than the measured one due to losses caused by the work performed with the gas during the foaming process.

### 2.6. Foam Morphology and Structure Characterization

SEM was used to observe the morphology of the foamed sample. The foam was cut by a ultrathin blade directly, without fracturing in liquid nitrogen, and then coated with Au before analysis. The cell size was determined by measuring the maximum diameter of each cell, and the cell density ($N_{n}$) was determined as follows:

(2) Nn=nA23φ

where n represents the counted cell number on a corresponding area A of the achieved image, and φ is the expansion ratio of the foamed sample, which can be calculated using the following equation:

(3) $$\varphi =\frac{\rho_{p}}{\rho_{f}}$$

where $\rho_{p}$ is the density of unfoamed polymer blend (g/cm^3^) and $\rho_{f}$ is the density of the foam sample (g/cm^3^).

Meanwhile, density tracking was performed to study the shrinkage behavior of the foam. The shrinkage ratio (S) was calculated by the following equation:

(4) $$S=\frac{\varphi_{0}-\varphi_{t}}{\varphi_{0}}\times 100\%$$

The expansion ratio at the initial time is recorded as $\varphi_{0}$, and the expansion ratio after stabilization is recorded as $\varphi_{t}$.

### 2.7. Mechanical and Thermal Properties

The shore C hardness of the prepared PBAT/PBS blend-modified foam was measured by a hardness tester (LX-C, Hongri Instrument Equipment Co., LTD., Fuding, China) according to ASTM D2240 [32]. The resilience was tested according to ASTM D2632 [33] using a digital-display falling-ball rebound tester from Hongri Instrument Equipment Co., LTD.

Compression set test (CS) was performed according to ASTM D395 [34], where foam samples were blanked into cylinders with a diameter of 3 cm, tested at 50 °C, 6 h, and a compression rate of 50%. At the same time, the samples were cut into rectangular block foams of 5 × 5 cm to test the thermal dimensional change (TD), and the test condition was 75 °C for 40 min. The calculation formulas are as follows:

(5) $$CS=\frac{d_{i}-d_{f}}{{d_{i}-d}_{0}}\times 100\%$$

where $d_{i}$, $d_{f}$, and $d_{0}$ are the initial thickness (mm), final thickness (mm), and compressed thickness (mm), respectively.

(6) $$TD=\frac{L_{i}-L_{f}}{L_{i}}\times 100\%$$

where $L_{i}$ is the initial length (cm), and $L_{f}$ is the final length (cm).

## 3. Results and Discussion

### 3.1. Compatibility of PBAT/PBS Blends

**Figure 2:**
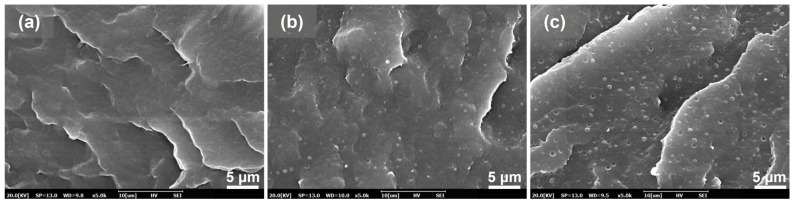
SEM images of the cryogenic fractured surfaces of the PBAT/PBS blends: (**a**) PBAT0; (**b**) PBAT5; and (**c**) PBAT10.

Compatibility is an important factor that affects the foaming behavior of polymer blends and properties of as-obtained foams. Figure 2 shows the cross-sectional morphologies of the PBAT/PBS blends. It can be seen that the pure PBAT is smooth and flat, showing a continuous single phase. After blending with PBS, the cross-section of PBAT/PBS blend shows a typical “see-island” two-phase structure. PBS is embedded in the PBAT phase in the form of spheres, and there is a small interfacial gap between the two phases. With the increase in the PBS content from 5 wt% to 10 wt%, the number of island phases gradually increased, and the phase size increased from about 0.4 μm to about 0.8 μm. According to the drop breakup theory in polymer blends that was proposed by Wu, the diameter of the droplet of the dispersed phase *D* can be determined by Equation (5) [35]:

(7) $$D=\frac{4\alpha p^{\pm 0.84}}{\dot{\gamma }\eta_{m}}$$

where α is the interfacial tension, $\dot{\gamma }$ is the shear rate, $\eta_{m}$ is the viscosity of main matrix, and p is the viscosity of the dispersed phase. The plus (+) sign applies for p > 1, and the minus (−) sign for p < 1. The interfacial tension between PABT and PBS has a small value of 1.38 mN/m [36]. Thus, the presence of micron-sized phases may be due to the shear rate of the extruder screw and the difference in melt index between PBAT and PBS.

### 3.2. Non-Isothermal Crystallization and Subsequent Melting Behavior

**Figure 3:**
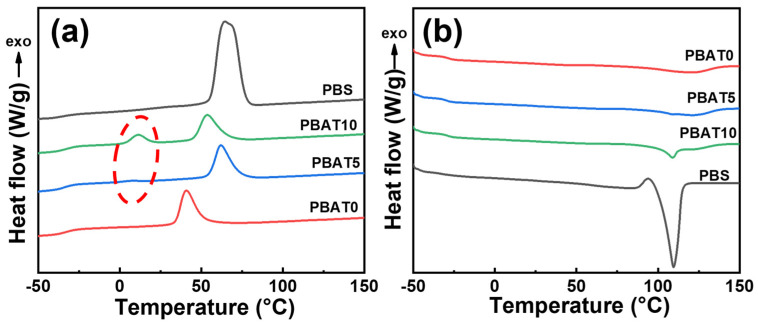
DSC thermal curves of PBAT/PBS blends: (**a**) cooling curves at a cooling rate of 10 °C/min; (**b**) second heating curves at a heating rate of 10 °C/min.

In order to study the thermal properties of PBAT/PBS blends, the non-isothermal crystallization and melting behavior were measured by DSC, and the results are shown in Figure 3. The relevant thermal properties data are listed in Table 1.

Figure 3a illustrates the cooling profiles of pure PBAT, pure PBS, and PBAT/PBS blends. It can be seen that both PBAT and PBS exhibit a single crystallization peak (*T*_c_). However, after blending with PBS, the PBAT/PBS blends exhibited two distinct crystallization temperatures (*T*_c_) (as shown by the red dotted circle). The second crystallization temperature (*T*_c2_) was higher than the first crystallization temperature (*T*_c1_) of pure PBAT, which is 40.8 °C, but lower than that of pure PBS, which is 64.5 °C. As a crystalline plastic, PBS exhibited high crystallization ability. The presence of near-nano-sized PBS phases might provide numerous nucleation sites, enhancing the crystallization of PBAT/PBS blends at a lower temperature. A similar phenomenon has been reported by others [37], and the similar chemical structures of PBAT and PBS were thought to be a critical reason for inhibiting the perfection of the crystalline domains in the PBS phase.

Figure 3b shows the secondary heating curves of PBAT/PBS blends with different ratios of PBS. Pure PBAT has one melting peak, with a wide melting range, suggesting a broad distribution of crystallite sizes and a low number of small crystallites. After the introduction of PBS, in addition to the “broad-shouldered” melting peaks (*T*_m2_) belonging to PBAT, sharp melting peaks (*T*_m1_), corresponding to PBS, gradually appeared in the PBAT/PBS blends. Furthermore, the crystallinity of the blends increased from 12.5% for PBAT0 to 17.6% for PBAT10. Higher crystallinity is beneficial in maintaining the thermal stability of the polymer.

### 3.3. Gas Solubility and Diffusivity

**Figure 4:**
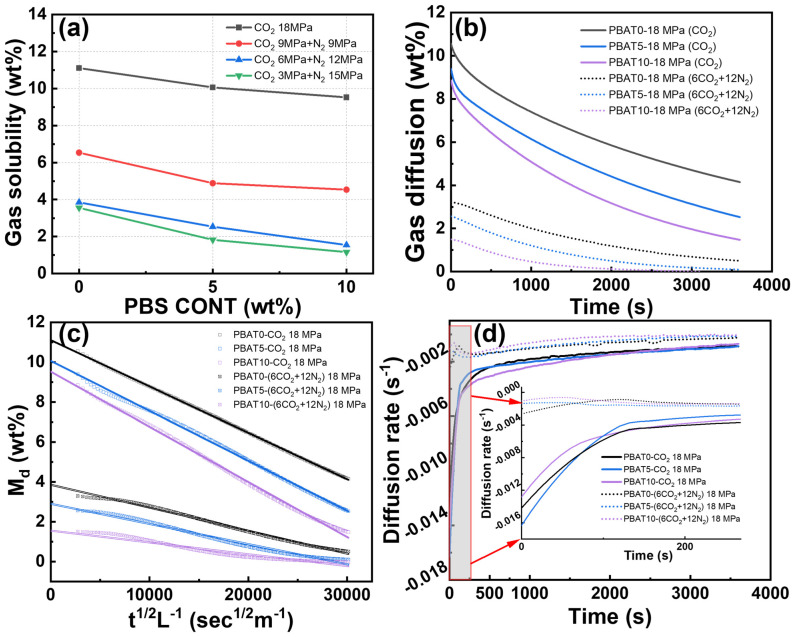
Gas dissolution and escape in PBAT/PBS blends. (**a**) Gas solubility of CO_2_ and CO_2_/N_2_ in polymer matrix; (**b**) residue of gas as a function of the desorption time; (**c**) extrapolation by linear fitting of the Fickian diffusion equation; (**d**) gas diffusion rate in PBAT with different gas mixtures.

During the physical foaming process of soft elastomers, the solubility and diffusivity rate of gases have critical influences on the expansion ratio and cell structure of foams. Figure 4a presents the gas solubility of the PBAT/PBS blends in pure CO_2_ and mixed CO_2_/N_2_ gases. The values are calculated and extrapolated from the one-dimensional Fickian diffusion equation (the linear fit extrapolation is shown in Figure 4c):

(8) $$\frac{M_{d}}{M_{\infty }}=1-\frac{4}{L}\sqrt{\frac{D_{d}t}{\pi }}$$

where $M_{d}$ is the mass uptake of samples at any time t, and $M_{\infty }$ refers to the solubility obtained by linear extrapolation, based on the desorption curve. $D_{d}$ denotes the desorption coefficient of gases, and *L* is the thickness of samples.

As indicated in Figure 4a, the gas solubility of CO_2_ or mixed CO_2_/N_2_ in PBAT blends decreased gradually with increasing PBS content, which was attributed to the increased crystallinity of the blends, since PBS exhibited high crystallization ability, and gas cannot dissolve within the crystalline domain. The applied gas pressure was fixed at 18 MPa in this study.Pure CO_2_ exhibited a high gas solubility of 10.6% for PBAT0, 9.4% for PBAT5, and 8.9% for PBAT10, respectively. The introduction of N_2_ tended to reduce the total gas solubility significantly, and a further increase in the N_2_ pressure led to the gas reduction. For instance, the solubility of PBAT5 decreased from 9.4% (CO_2_ 18 MPa + N_2_ 0 MPa) to 1.8% (CO_2_ 3 MPa + N_2_ 15 MPa). This is attributed to the fact that CO_2_ solubility is much higher than that of N_2_. A similar phenomenon has been widely reported in PEBA and LDPE foaming systems [9,38,39].

Figure 4b depicts the gas diffusivity process in the sample over a 1 h period. The first-order derivative of this plot yields the gas diffusivity rate, as illustrated in Figure 4d [40]. Pure CO_2_-saturated sample exhibits a significantly high gas diffusivity rate in the initial 200 s, reaching a maximum value of−0.016/s. Conversely, the gas in the gas mixed CO_2_/N_2_ saturated sample consistently escapes at a lower rate, which is one order of magnitude lower than pure CO_2_. It is noteworthy that the gas escape rate in the samples decreases slightly, though not significantly, with the addition of PBS.

### 3.4. Foaming Behaviors of PBAT/PBS Blends Blown with CO_2_

**Figure 5:**
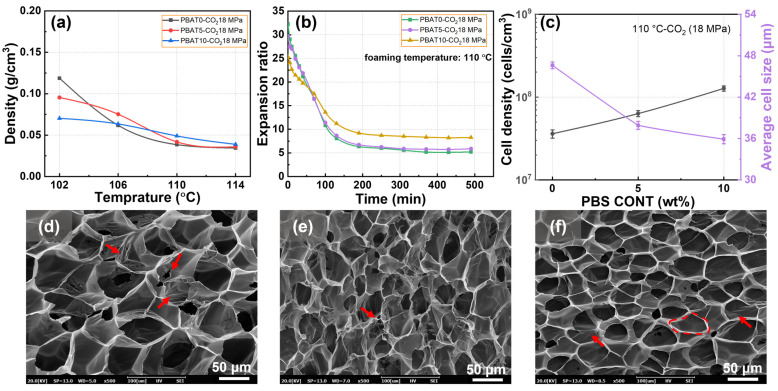
Foaming and shrinkage behavior of PBAT/PBS blends blown with pure CO_2_: (**a**) density of initial PBAT and PBAT/PBS foams prepared at various foaming temperatures; (**b**) shrinkage behavior of PBAT and PBAT/PBS foams over time; (**c**) cell size and cell density of PBAT and PBAT/PBS foams prepared at a foaming temperature of 110 °C; (**d**–**f**) SEM micrographs of PBAT and PBAT/PBS foams prepared at a foaming temperature of 110 °C.

The foaming behavior of PBAT and PBAT/PBS blown with CO_2_ was investigated, and the results of foam expansion and shrinkage are shown in Figure 5. It can be seen that, after being foamed by pure CO_2_, both the PBAT and PBAT/PBS blends presented extremely low initial foam densities of about 0.04–0.05 g/cm^3^ at the foaming temperature of 110–114 °C (Figure 5a), which was associated with a 25–31 times volume expansion during the foaming process. When the initial foams were aged under atmospheric pressure, as indicated in Figure 5b, the expansion ratio of the initial foams tended to decrease dramatically within 130 min and then level off at the extended aging time. Relative to the initial expansion ratio, the aging process led to a rough 67–81% density increment. The obvious foam shrinkage of elastomer foams has been widely observed, which is attributed to the fact that the low elastic modulus of elastomer cannot resist the pressure difference inside and outside the cell wall caused by the rapid gas escape [41].

**Figure 6:**
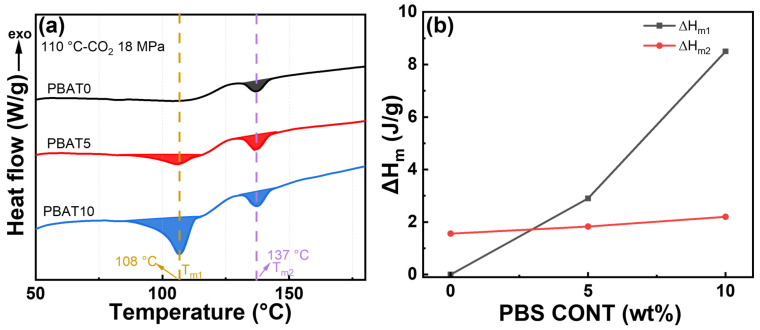
DSC thermograms of PBAT and PBAT/PBS foams: (**a**) first heating run of samples foamed at 110 °C-CO_2_ 18 MPa; (**b**) melt enthalpy of foaming samples.

Figure 5b–f illustrate the influences of PBS loading on the foam shrinkage behavior and cell morphologies of PBAT foams, with the foaming condition set at 18 MPa and 110 °C. Figure 5b shows that the PBS loading tends to decrease the initial expansion ratio of blend foam but increase the stable expansion ratio of blend foams. Figure 6 shows the melting behavior and Δ*H*_m_ of PBAT/PBS blend foams. Relative to PBAT foam, the increased Δ*H*_m1_ of PBAT5 and PBAT10 foams increased the matrix modulus, which prevented the initial foam expansion and prevented the shrinkage of foam during the aging process. The observed *T*_m2_ peak is likely due to the plasticizing influence of CO_2_, which promotes the reorganization of PBAT molecular chains into a more perfected crystalline arrangement, thus elevating the melting temperature [42].

The cell morphology presented in Figure 5d–f also indicates the contribution of PBS loading on foaming behavior of the PBAT/PBS blends. As indicated in Figure 5d, the PBAT0 foam presented a partially open cell structure (as shown by the arrows in the figure), which was because of the fracture of the cell wall, caused by the extremely high initial volume expansion. PBAT5 and PBAT10 exhibited intact cell structures, and the possible reasons were the decreased extensional force due to the reduced initial volume expansion and the increased matrix strength due to the presence of the crystallized PBS phase. Meanwhile, as shown in Figure 5c, PBS loading helped decrease cell size from 46.6 to 35.9 μm and increase cell density, which was contributed to the heterogeneous cell nucleation effect of the near-nano-sized PBS phase.

### 3.5. Foaming Behaviors of PBAT/PBS Blends with Mixed CO_2_/N_2_ Gas

**Figure 7:**
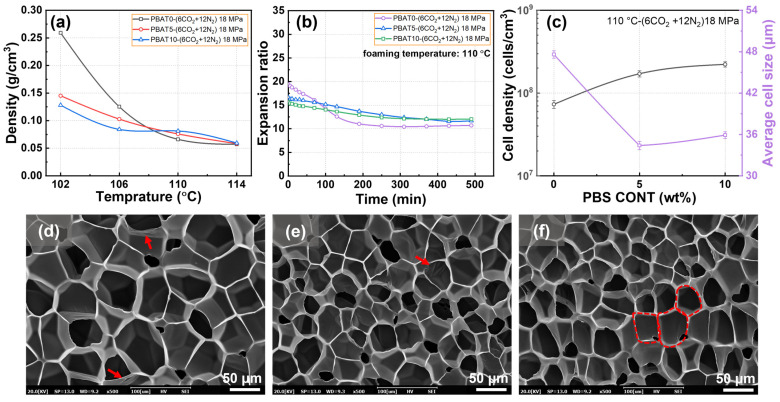
Foaming and shrinkage behavior of PBAT/PBS blends blown by the mixed CO_2_/N_2_: (**a**) density of PBAT and PBAT/PBS foams prepared at various foaming temperatures; (**b**) shrinkage of PBAT and PBAT/PBS foams as a function of aging time; (**c**) cell size and cell density of PBAT and PBAT/PBS foams prepared at the foaming temperature of 110 °C; (**d**–**f**) SEM micrographs of PBAT and PBAT/PBS foams.

Figure 7 illustrates the foaming and shrinkage behavior of PBAT/PBS blends blown by the mixed gas. The total pressure was 18 MPa, the partial pressure of CO_2_ was 6 MPa, and that of N_2_ was 12 MPa. As indicated in Figure 7a, the initial density of all three PBAT foams was about 0.057–0.081 g/cm^3^ at the foaming temperatures of 110–114 °C, which was associated with a volume expansion of about 15.3–21.8 times. Compared to pure CO_2_, the mixed CO_2_/N_2_ had lower solubility, which was a main reason for the lower initial volume expansion of the three PBAT foams. Figure 7b shows that the expansion ratio of PBAT foams decreases slightly with the aging treatment and levels off at the extending time of about 250–350 min. Relative to pure CO_2_, PBAT foams blown by the mixed CO_2_/N_2_ presented much smaller volume shrinkage during the aging process. The phenomenon was attributed to a lower gas diffusivity of N_2_ vs. CO_2_, as well as the smaller initial foam expansion. Furthermore, Figure 7b shows that the PBAT/PBS blend foams possess a lower shrinkage degree compared to the PBAT foam, which is attributed to the increased matrix modulus because of the dispersed PBS phase.

The cell morphology shown in Figure 7c–f and the cell parameters shown in Table 2 also present the contribution of the mixed gas and PBS loading on the foaming behavior of PBAT. An intact cell structure can be observed in all three PBAT foams, where no open cell structure was seen within the cell structure. The benefit of the PBS phase on the cell morphology can be verified in Figure 7c, where the decreased cell size and increased cell density was caused by the heterogeneous cell nucleation of the neari-nano-sized PBS phase.

**Figure 8:**
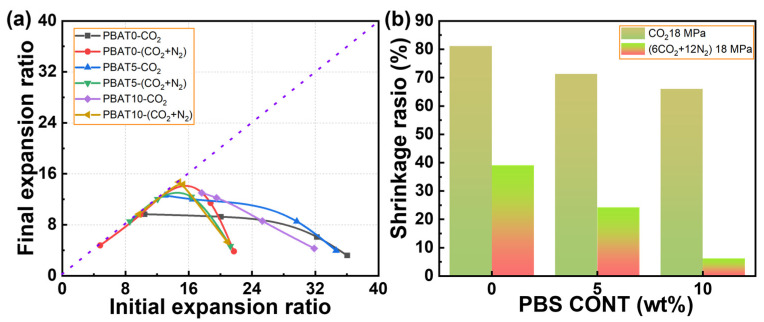
Degree of foam shrinkage of PBAT/PBS blends: (**a**) initial and final expansion ratio; (**b**) shrinkage ratio as a function of PBS content.

All data on volume shrinkage are presented in Figure 8, where the influences of initial expansion ratio, the usage of N_2_, and PBS loading on volume shrinkage are summarized. As indicated in Figure 8a, under fixed pressure and temperature, the maximum final expansion ratio of PBAT foams was about 14.7 times, and the increase in initial expansion ratio usually led to a low final expansion ratio. Figure 8b shows that the PBS loading always helps reduce the shrinkage ratio of PBAT foam, regardless of whether N_2_ is used, and the minimum shrinkage ratio of 6.1% was obtained with 10% PBS loading blown with the mixed CO_2_/N_2_.

**Figure 9:**
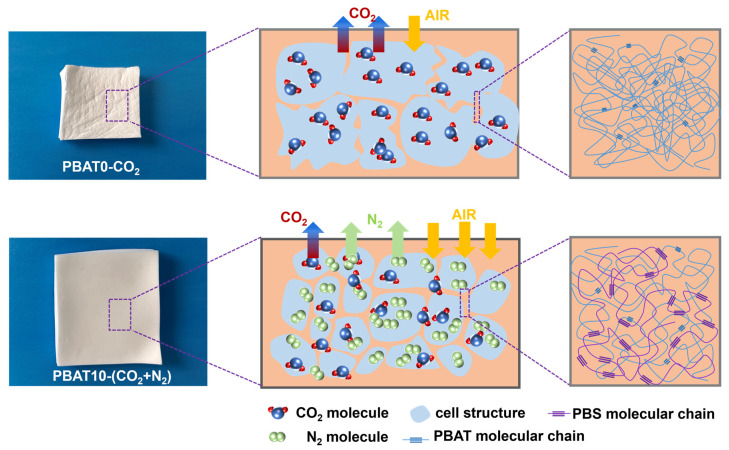
Schematic diagram of the shrinkage mechanism of PBAT foams.

A schematic diagram, shown in Figure 9, is used to illustrate the synergistic effect of N_2_ usage and PBS phase in inhibiting foam shrinkage. CO_2_ usually has a high solubility and high gas diffusivity within polymers, especially for polymers with polar groups [41]. A higher gas concentration tends to supply more space for cell growth to obtain a higher volume expansion, and a higher gas diffusivity tends to apply higher extensional force to the cell wall. The generated force during cell growth could break the cell wall and enhance gas escape out of the foam under high temperatures within the foam core. On the other hand, the air surrounding the foam at room temperature has low gas diffusivity, and it needs time to penetrate into the cell wall. In some conditions, the pressure difference within and outside the cell structure might be negative [23]. In the case of soft PBAT with lower crystallinity, the modulus of cell walls was low. Therefore, it might not withstand the pressure difference, and hence tend to shrink significantly, characterized by the wrinkled foam surface. The formation of open cells might supply more channel for gas to escape, which would accelerate foam shrinkage and lead to an increase in density. 

With the introduction of N_2_, the gas solubility and gas diffusivity tended to decrease, which facilitated the decrease in the generated force during cell growth and the formation of an intact cell structure. Meanwhile, the low gas escape rate would provide more time for air penetration into the cell structure, and the reduced pressure difference within and outside the cell wall could resist foam shrinkage. The benefit of nano-sized PBS phase in reducing foam shrinkage was caused by its high modulus because of its high crystallinity. Thanks for the PBS loading and N_2_ usage, we could fabricate a PBAT10 foam with low foam density and smooth foam surface.

### 3.6. Thermo-Mechanical Properties of the Foams of PBAT/PBS Blends

**Figure 10:**
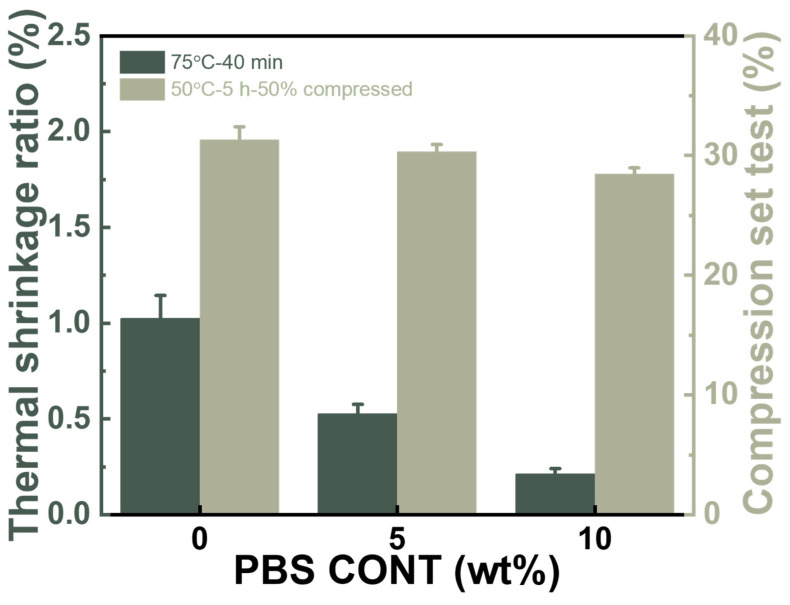
Thermal stability of PBAT and PBAT/PBS blend foams at 70 °C/40 min.

The non-crosslinking PBAT foam has the advantage of being bio-degradable, but it may suffer from its low thermal stability. In a real usage environment, a tolerance of 75 °C is usually required. Figure 10 shows the dimensional stability of PBAT foams with various PBS loading contents, and the testing condition of 75 °C/40 min for shoe application was applied. It can be seen that PBAT0 foam exhibits a 1.0% size shrinkage after the thermal treatment, and the presence of PBS reduced the shrinkage dramatically to 0.5% for PBAT5 and to 0.2% for PBAT10, due to the increased crystallinity of the blends. 

The compression set is a critical parameter for evaluating the plastic deformation of soft foam under a compression environment, and a reduced value means increased elasticity. It has been widely reported that the EVA foam with a density of 0.15–0.20 g/cm^3^ has a compression set of about 40–60% [43,44], while that of TPU foam with a density of 0.15 g/cm^3^ has a value of 25–35% [45,46]. Figure 10 shows that the compression sets of both PBAT and PBAT/PBS foams are 28.5–31.3%. This is similar to the TPU foam but much better than the cross-linked EVA foam. The excellent compression set value ensures that PBAT foam can be used in shoe foam applications. It was interesting to find that the PBS loading decreased the compression set a little bit, which suggests that the nano-sized PBS phase did not increase plastic deformation under compression.

### 3.7. Resilience and Hardness of the Foams of PBAT/PBS Blends

**Figure 11:**
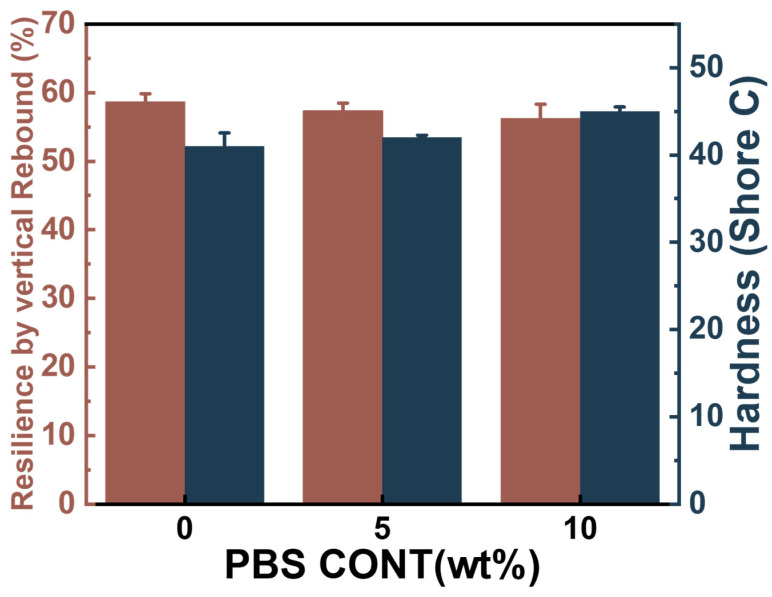
Resilience and hardness of PBAT/PBS blend foams with different PBAT/PBS blending contents.

Besides the compression set, the elasticity performance is another critical parameter to evaluate the soft foam. A falling-ball rebound tester was used to calculate the elasticity of the PBAT foams, and the results are shown in Figure 11. It is indicated that the resilience of the PBAT foams is about 56.2–58.7%, which is higher than that of the EVA foam, with a value of about 50% [47], but is similar to that of aromatic TPU foam, with a value of about 55–60% [45]. This demonstrates that the PBAT foam with non-crosslinking structure already presents well-defined elasticity, making it a potential candidate for replacing EVA foam or even TPU foam in shoe applications. 

Figure 11 indicates that PBAT foams have a hardness of about 41–45 degree, which is the usual requirement for EVA foam in shoe applications. Meanwhile, the small amount of PBS loading did not obviously increase the hardness of foams, ensuring that the PBAT/PBS blend foam has an excellent surface feel. 

## 4. Conclusions

In this work, soft PBAT and PBAT/PBS blend foams were prepared by a physical foaming process. Both CO_2_ and a CO_2_/N_2_ gas mixture were used as blowing agents. PBAT and PBS had similar chemical structures, and the near-nano-sized tiny PBS phase could be observed in PBAT5 and PBAT10. CO_2_ presented high gas solubility and high gas diffusivity, which ensured the as-prepared PBAT foam with a 31 times initial volume expansion, but extreme shrinkage only caused a 5.7 times expansion in the final foam. This kind of shrinkage could be significantly mitigated by using a CO_2_/N_2_ mixture, where the mixed gas presented much lower gas solubility and gas diffusivity. The influence of PBS and N_2_ usage on the foam shrinkage was investigated and then optimized, and the PBAT10 foam, with maximum expansion ratio of 14.7 times, was achieved. A schematic diagram was built to explain the possible mechanism. The decrease in pressure difference within and outside the cell structure and the increased modulus due to the loading of the crystallized PBS phase are possible reasons for the preparation of PBAT/PBS foams with a higher expansion ratio, less shrinkage, and smooth foam surface. The properties of PBAT/PBS foams were investigated, and the foams, with suitable hardness, low compression set, and high rebound, were found to be useful for shoe applications. The PBS loading of 5–10% did not spoil the soft nature of the PBAT foam but improved its thermal stability, which is beneficial for its use in real-world applications.

## Figures and Tables

**Table 1:** Thermal parameters of pure PBAT, PBAT/PBS blends, and PBS.

Sample	*T*_c1_ (°C)	Δ*H*_c1_ (J/g)	*T*_c2_ (°C)	Δ*H*_c2_ (J/g)	*T*_m1_ (°C)	*T*_m2_ (°C)	Δ*H*_m_ (J/g)	*X*_c_ (%)
PBAT0	40.8	19.54	-	-	120.0	-	14.2	12.5
PBAT5	3.5	1.3	62.0	19.68	108.0	123.2	16.06	14.1
PBAT10	11.4	4.3	53.6	16.76	109.0	124.1	20.04	17.6
PBS	64.5	60.0	-	-	109.6	-	51.6	46.8

**Table 2:** Statistical data on the structure of cells at different gas ratios and PBS contents.

Samples	Gas Ratio (CO_2_:N_2_) (MPa)	Cell Size (μm)	Cell Density (Cells/cm^3^)
PBAT0	18:0	46.6	3.6 × 10^7^
	9:9	45.3	8.8 × 10^7^
	6:12	47.6	7.3 × 10^7^
	3:15	45.8	1.1 × 10^8^
PBAT5	18:0	37.9	6.3 × 10^7^
	9:9	36.8	7.8 × 10^7^
	6:12	34.4	1.7 × 10^8^
	3:15	32.7	3.8 × 10^8^
PBAT10	18:0	35.9	1.3 × 10^8^
	9:9	34.6	3.6 × 10^8^
	6:12	35.9	2.2 × 10^8^
	3:15	33.1	3.8 × 10^8^

## Data Availability

The original contributions presented in the study are included in the article, further inquiries can be directed to the corresponding author.
